# Effectiveness of Scaling up the ‘Three Pillars’ Approach to Accelerating MDG 4 Progress in Ethiopia

**Published:** 2014-12

**Authors:** Mary A. Carnell, Leanne Dougherty, Amanda M. Pomeroy, Ali M. Karim, Yared M. Mekonnen, Brian E. Mulligan

**Affiliations:** ^1^John Snow, Inc., Arlington, VA, USA; ^2^Mela Research, PLC, Addis Ababa, Ethiopia; ^3^Bill & Melinda Gates Foundation, Seattle, WA, USA

**Keywords:** Child health, Community, Evaluation, Health system strengthening, Scale up, Ethiopia

## Abstract

This paper describes the integrated approach taken by the Government of Ethiopia with support from the Essential Services for Health in Ethiopia (ESHE) Project and assesses its effect on the coverage of six child health practices associated with reducing child mortality. The ESHE Project was designed to contribute to reducing high child mortality rates at scale among 14.5 million people through the ‘three pillars’ approach. This approach aimed to (i) strengthen health systems, (ii) improve health workers’ performance, and (iii) engage the community. The intervention was designed with national and subnational stakeholders’ input. To measure the Project's effect on the coverage of child health practices, we used a quasi-experimental design, with representative household survey data from the three most populous regions of Ethiopia, collected at the 2003-2004 baseline and 2008 endline surveys of the Project. A difference-in-differences analysis model detected an absolute effect of the ESHE intervention of 8.4% points for DTP3 coverage (p=0.007), 12.9% points for measles vaccination coverage (p<0.001), 12.6% points for latrines (p=0.002), and 9.8% points for vitamin A supplementation (p<0.001) across the ESHE-intervention districts (*woredas*) compared to all non-ESHE districts of the same three regions. Improvements in the use of modern family planning methods and exclusive breastfeeding were not significant. Important regional variations are discussed. ESHE was one of several partners of the Ministry of Health whose combined efforts led to accelerated progress in the coverage of child health practices.

## INTRODUCTION

Ethiopia faced a dire child health situation when the Millennium Development Goals (MDGs) were set, ranking sixth globally in the number of under-five deaths in 2000 (1). One-half of the surviving under-five children suffered from moderate to severe undernutrition (2). In 2007, the first-year data were available, only 0.25 health workers (physicians, nurses, and midwives) per 1,000 population were present, i.e. one-tenth of the minimum threshold set by the World Health Organization (3-4). Health facilities were few and far between; approximately 50% of the population resided over 10 km from a health centre (5).

The Ethiopian Federal Ministry of Health (FMOH) invested in an ambitious national programme to increase resources to address high mortality rates. The Essential Services for Health in Ethiopia (ESHE) Project was designed to complement this effort with the ‘three pillars’ strategy to improving child health practices at scale in the three largest regions, covering 14.5 million people. The three pillars included (i) strengthening health systems, (ii) improving health workers’ performance, and (iii) engaging the community. The ‘three pillars’ approach was based on the WHO's Integrated Management of Childhood Illness (IMCI) framework but was applied more broadly, looking beyond the ‘sick child’ contact with the health system, which is central to IMCI. While IMCI has been effective in addressing child morbidity, it has shown limited success in increasing the coverage of preventive child health interventions (6-7). Evidence on the impact of IMCI often focuses on the impact of health workers’ training opposed to the impact of an integrated approach, including health system strengthening and community engagement (8).

In 2003, a series of articles on child survival published in *The Lancet* described a set of evidence-based preventive and curative interventions with high impact on child mortality reductions (1,9-12). Exclusive breastfeeding (EBF), management of diarrhoea, sanitation and hygiene, immunization, and malaria case management, among others, were determined to have sufficient evidence of benefit in efficacy and effectiveness, and these were recommended (11). This series of articles corroborated the ESHE strategy that included several of these evidence-based interventions: Expanded Programme on Immunization (EPI), IMCI (diarrhoea, pneumonia, malaria, measles, and malnutrition), and Essential Nutrition Actions (ENA) (breastfeeding, complementary feeding, vitamin A and iron/folic acid supplementation).

*The Lancet* series both confirmed that evidence-based interventions decrease child deaths and called for further evidence on how to package, phase-in, and implement interventions to achieve the coverage at scale to measurably impact child mortality rates. Literature had focused on documenting the effects of community-level health promoters in improving the coverage of key child health interventions (13-16). The Cochrane review of lay health workers’ impact identified immunization and breastfeeding as interventions with a ‘strong’ level of evidence for effectiveness of volunteers to improve effect (17). Only a few programmes have implemented the integrated ‘three pillars’ approach. In Madagascar, the Basic Support for Institutionalizing Child Survival Project (BASICS) demonstrated significant increase in the coverage in several evidence-based child health interventions (18-19). Increases in breastfeeding, using a similar ‘three pillars’ approach, were documented in Latin America and Africa (20-21).

The purpose of this paper is to describe how ESHE implemented the ‘three pillars’ approach and to provide evidence on the plausible effect of this integrated approach in improving the coverage of six child health practices at scale.

### Background and description of the intervention

#### Ethiopian health system context

Two complementary government initiatives were considered when designing ESHE: (i) accelerated expansion of primary healthcare coverage in Ethiopia (2005-2009) and (ii) implementation of the Health Extension Worker Program (HEP). The first initiative created population-based primary healthcare units (a health centre and five health posts) to focus on increasing quality of care to serve 25,000 people each. The second initiative added over 30,000 women health extension workers (HEWs) based in 15,000 health posts (22-23). Two HEWs were deployed in each *kebele*, the lowest administrative unit, to provide 16 service packages (24-25). HEP includes promotive, preventive and limited curative health services in 4 areas totaling 16 service packages: (i) hygiene and environmental sanitation: proper and safe excreta disposal system, proper and safe solid and liquid waste management, water supply safety measures, food hygiene and safety measures; healthy home environment, arthropods and rodent control, and personal hygiene; (ii) disease prevention and control: HIV/AIDS prevention and control, TB prevention and control, malaria prevention and control, and first aid; (iii) family health services: maternal and child health, family planning, immunization, adolescents’ reproductive health, and nutrition; and (iv) health education and communication.

HEWs focused primarily on preventive and promotive services at the community level and linking communities to health centres for most curative care services (26,27).

#### Description of ESHE's intervention

The ESHE Project was designed with FMOH stakeholders to strengthen the newly-decentralizing health system and to accelerate the coverage of child health practices by reinforcing these two complementary government initiatives. USAID could not support work in all *woredas*; therefore, selection criteria for intervention included high population density, low to moderate past programme performance [antenatal care coverage, diptheria/tetanus/pertussis3 (DTP3) coverage, modern contraceptive prevalence rate (MCPR)], and presence of other health partners. Discussion with regional health bureaus first identified zones (group of 8-10 *woredas*). Final selection of *woredas* was a compromise, including some difficult-to-reach, poor-performing zones and some easier-to-access, moderate-performing zones. Within the zones, a cluster of 5 *woredas* was selected to make it more efficient to provide technical assistance. The comparison group comprised all *woredas* not selected for intervention in the three regions.

**Figure 1.**
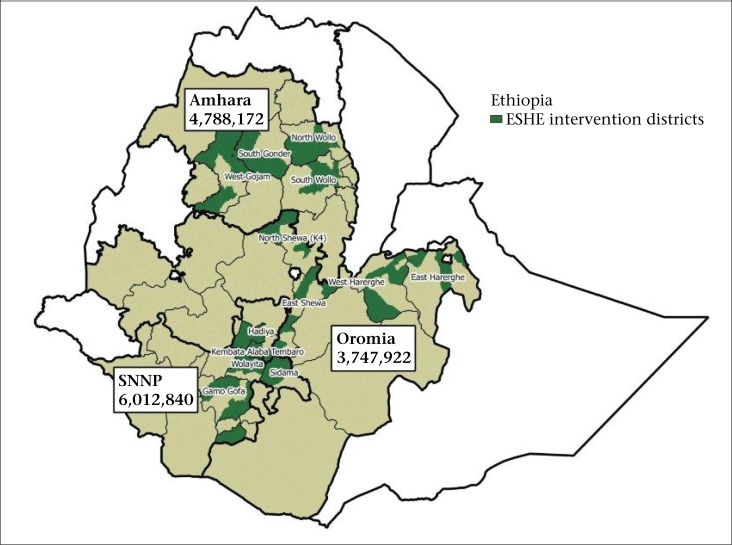
Map of ESHE intervention districts and population targeted in three regions

The ESHE Project began in early 2004 in 24 out of 60 *woredas* of Southern Nations, Nationalities and Peoples’ (SNNP) region. ESHE's interventions expanded in 2005 to Oromia (20 *woredas* of 133) and Amhara regions (20 *woredas* of 83) (Figure 1).

The total programme cost was US$ 23 million, or US$ 1.60 per capita over five years. The ESHE Project teams were housed, when possible, at regional and subregional (zonal) health offices. Strategic co-location permitted daily contact with government counterparts and enhanced capacity building, communication, ownership and sustainability of project interventions. Two ESHE technical staff members were added to the *woreda* management team. They were equipped with one vehicle and driver and were located in zonal offices to assist an average of five rural *woredas*. In 2003, the approach to mobilizing community health promoters (CHPs) to implement an integrated package of child health interventions at scale was unique to the ESHE districts. In July 2005, HEWs were directed to create a cadre of volunteer CHPs when deployed as part of HEP. The Third Health Sector Development Plan 2005-2010 endorsed the creation of voluntary community health promoters with one CHP per 50 households under the coordination and supervision by HEWs. The ESHE had already begun implementing a CHP initiative and provided a systematic method to implement this directive.

The CHP initiative was designed to enhance the reach of two HEWs in each *kebele*. The process was informed by seven years of similar programme experience in Madagascar where volunteers were selected by the community following a broad sensitization meeting (18-19,21). Twenty to thirty volunteers were selected per *kebele* based on an average population of 5,000 in each *kebele*. Early ESHE experience informed the FMOH policy for a 1:10 ratio of HEWs:Volunteers. The volunteers, approximately 50% being female, were geographically spread throughout the *kebele*. Literacy was not a requirement. The CHPs were initially trained to negotiate with neighbours about taking key health actions for their children and family members.

**Figure 2.**
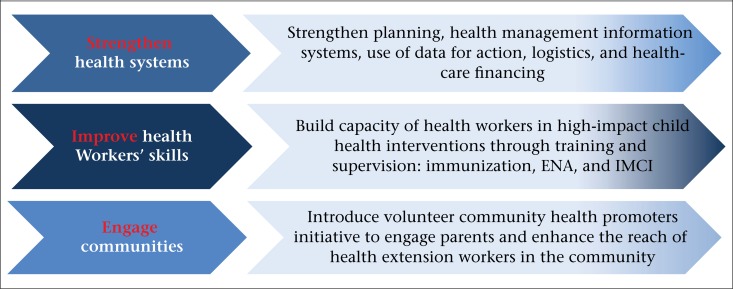
Summary of ESHE support to district-level health system

HEWs in the ESHE-supported *woredas* received training in child health and community health promotion. HEWs received BCC materials and were trained to conduct volunteer review meetings. Although ESHE engaged at the national level on child health policy and healthcare financing reform, it focused on selected *woredas* in the three regions. Figure 2 summarizes the ‘three pillars’ approach used for support to the *woredas*.

### Pillar 1: Strengthen health systems

The intervention aimed to strengthen *woreda-* and facility-level management through improved health management information systems (HMIS) and increased use of data for decision-making [See ESHE training in Table 1].

HMIS review teams were established in all health facilities and *woreda* health offices to review data monthly and create action plans to improve outcomes (5). Examples of actions taken include HMIS data cleaning to improve information quality, identifying bottlenecks and seeking solutions for low programme performance, and updating performance monitoring wall charts. Supervision included monitoring whether these wall charts were updated and minutes of the HMIS review team meeting were available.

*Woreda* health management teams were trained in healthcare financing and management in four-day courses. Starting in 2006, *woreda* staff received on-the-job training by ESHE staff during decentralized annual planning exercises.

Monthly review meetings at *woredas* and health facilities brought staff together to review achievements, share good practices, and agree on strategies to overcoming bottlenecks. The Project provided technical and some financial assistance for supervision and quarterly reviews at zonal and regional levels.

**Table 1.** Training, by number of personnel trained, days of training, and year of the Project

Training	No. trained	No. of days	Year
HMIS and data for decision-making	136 managers	4	One
Supportive supervision	847 managers	4	Two
Healthcare financing and management	4,765 managers and health workers	4	Three
Immunization	1,148 health workers and HEWs	4	One
Essential Nutrition Actions	1,624 health workers and HEWs	4	Two
IMNCI*	618 health workers	6	Three
Community health promoters' TOT	6,055 health workers and HEWs	2	One-three
CHP first round (EBF, EPI, water/sanitation)	54,582 community health promoters	2	One-three

*Revisions in HEW policy and protocols were required before HEWs could be trained in IMNCI; these revisions occurred in 2010 after the ESHE ended

### Pillar 2: Improve health workers' performance

#### Child health technical training

The intervention aimed to increase the capacity of FMOH managers and frontline health workers (facility-based workers and HEWs) in three child health programmes: EPI, ENA, and IMNCI. IMCI was revised in Ethiopia in 2004 to include essential newborn care and care of the sick newborn. The name was changed to the integrated management of newborn and childhood illnesses (IMNCI). In collaboration with the FMOH and regional health bureaus (RHBs), training materials were adapted to the Ethiopian context. The Project staff assisted regions to implement training-of-trainers and *woreda-*level training courses. Scaling up was phased-in by introducing one intervention each year [See Table 1 for training provided]. All training courses were competency-based.

#### Supportive supervision and performance improvement

The intervention increased the frequency and quality of the supervision of health workers and included development of management and service delivery standards. Following the training, ESHE supported tailored ‘follow-up to training’ visits within six weeks and quarterly integrated supportive supervisory visits. The Project staff assisted *woreda* counterparts to conduct these visits. An integrated supervisory checklist was developed with regional and *woreda-*level staff.

### Pillar 3: Engage communities to improve community and household health practices

#### CHP initiative

*Woreda,* health facility, and ESHE staff led community sensitization meetings to introduce the CHP initiative. The same staff facilitated volunteer training and supervision after *kebeles* selected their CHPs. When HEWs were deployed, ESHE quickly transitioned to make HEWs the focal point of the CHP programme. HEWs were trained to sensitize community leaders and to assist the *kebele* in selecting, training, and supervising volunteers. This transition decreased cost by decentralizing training and increased the speed of scaling up, local ownership, and sustainability. Over the intervention period, 54,582 CHPs were trained on immunization, breastfeeding, water/sanitation, and interventions included in the community IMCI strategy. Additional training courses on other child health practices were introduced once CHPs were confident on promoting the first set of actions [See Table 1 for CHP training courses].

An integral component of the CHP initiative was support and encouragement to volunteers. Review meetings were conducted three months after training. HEWs led monthly meetings with volunteers in their communities to review efforts and add new themes, gradually building the CHP's capacity. Non-financial incentives were employed to motivate volunteers, including annual festivals to recognize voluntary efforts within the community. More details on the interventions are available elsewhere (28).

## MATERIALS AND METHODS

We used a before-after design of cross-sectional representative household surveys conducted in 2003-2004 and 2008 from ESHE and non-ESHE areas and measured plausible programme attribution by comparing average changes in the child health practices of interest between baseline and follow-up surveys in the ESHE areas with the changes in the non-ESHE areas.

### Study design

In 2003-2004, baseline data were collected or constructed in the three regions to be used in conjunction with an endline household survey for planning, monitoring, and final evaluation. The household surveys were administered according to a two-stage WHO's EPI cluster-sampling methodology, which divided each region into two sampling domains for the entire region: *woredas* with the ESHE Project focus and non-project *woredas* (29-30). Weighting results from these two domains provided the RHBs with a population-based estimate of regional performance between the five-year DHS surveys. Thirty clusters were randomly selected from each domain, resulting in approximately 60 clusters per region. *Kebeles* were used for delineating the clusters. The ESHE domain accounted for more than 14.5 million people in *woredas* with project focus, and the non-project domain accounted for the remaining 32.6 million people across the three regions (31).

Sample-size calculations were adopted from WHO's EPI sampling methodology, and clusters included 10 women aged 15-49 years, 10 women reporting on children aged 0-11 month(s), and 10 women reporting on children aged 12-23 months. These groups were selected to provide responses regarding the various ESHE objectives. This method of calculation is assumed to provide confidence limits which have been shown not to exceed ±10 absolute percentage points in a cross-sectional survey (29). To examine the power of our study, we conducted a post-hoc power analysis.

In the final year of the Project (2008), endline surveys were conducted to assess progress following the same sampling methodology. In total, 3,900 women, 3,929 children aged 0-11 month(s), and 3,904 children aged 12-23 months were included. Details of survey design, questionnaire, training, supervision, data management/quality control, and dates of data collection are reported elsewhere (32-34). Missing values at endline represented less than 2% of the total sample and were censored from the analysis.

Because SNNP had been the site of an earlier child health project with an endline survey conducted immediately prior to ESHE, a baseline survey comparable to the other two regions was designed from this endline data. SNNP baseline was established for ESHE intervention and comparison areas, using all clusters available in the previous endline survey with appropriate weights assigned to each cluster. Data from 43 clusters of the previous project endline survey fell into the current ESHE project intervention area. The 43 clusters were stratified by district and the following population weighting applied: P1/p2 where P1 represents the proportion of the total population of ESHE intervention areas represented by each district and p2 is the proportion of respondents represented by each cluster. As a result, the SNNP baseline had more clusters and slightly more children than in the other two regions and periods. The ESHE Project included new *woredas* and some *woredas* from endline of the earlier child health project. Table 2 presents the sample-size for each region and for the total ESHE sample by period and group.

### Key child health practices

To measure the effect of ESHE interventions, the following two key practices were assessed for each of the three child health programmes implemented:

*EPI*: DTP3 and measles vaccination coverage were collected from mothers of children aged 12 to 23 months.

*Community IMCI:* Household latrine ownership and the use of modern contraceptive methods were collected from women aged 15 to 49 years.

*ENA:* EBF was collected from mothers of children aged 0 to 5 month(s); vitamin A supplementation in the past 6 months, from mothers of children aged 6-23 months.

Data collection and variable construction followed global standard questions and sequences used by WHO/UNICEF/DHS/MICS for EPI, water and sanitation, vitamin A, modern family planning, and EBF. These indicators were selected because these were government priorities and represented key evidence-based practices for child survival. Complete household survey reports with additional indicators are available elsewhere (32-35).

### Data processing and analysis

Because the ESHE baseline surveys followed a modified WHO's EPI method, respondents for the women's survey were selected for household characteristics (such as latrines and water source), irrespective of whether they were linked to the surveyed children. When linking women's data to the children's data for analysis, approximately half of the children did not have an interviewed mother, resulting in missing sociodemographic characteristics for those children. Endline surveys corrected this problem.

Since mother's education and, to a lesser extent, a household's time/distance to the health facility, have been shown to have a significant effect on DTP3 coverage and EBF (36-38), it was necessary to impute data to control for these variables in our analysis. For children without a linked mother in their baseline, we used *kebele*-level cluster means for all women, not just mothers, to impute the data, assuming that women's time to a health facility and also educational level will co-vary somewhat with their neighbours. This is plausible because our sampling methodology targeted a subarea within a *kebele*, leading to a less geographically-dispersed sample. Because there are only three categories of classification for each variable, it was considered prudent to assume that time to health centre [less than one hour, one to two hour(s), or over two hours] and education (no education, primary, or more than primary) could be generalized across a cluster. A significant majority of women had no education, lessening variation in this variable. To check sensitivity of results to the inclusion of these data, we tabulated outcomes of children with complete data and those missing sociodemographic characteristics for the child and found minimal differences.

The following logistic regression equation was used for measuring ESHE effect:

Logit (Y_i_) = β_0_ + β_1_ (T_i_) + β_2_ (t_i_) + β_3_ (T^x^ t_i_) + β_4_ (c_i_) … + ϵ_i_,

where Y=binary coded outcome of interest, T=ESHE participation (coded ‘1’ for respondents in the ESHE areas and ‘0’ for those in the non-ESHE areas), t=time period (coded ‘1’ for follow-up and ‘0’ for baseline), ϵ=error of the model or unexplained variance, i=respondents and c=other control variables. Control variables in the woman's model included age, educational level, distance to health facility, and region. Control variables in the child models included sex of the child, region, time to health facility, and maternal education. The relative change (i.e. odds ratio) in the outcome of interest in the comparison area is estimated by exp (β_1_) while the relative change in the intervention area is estimated by exp (β_1_+β_3_). Therefore, the difference-in-differences effect is given by exp (β_3_) (the difference in the changes over time in the probability of a child health outcome in the non-ESHE areas from changes in the ESHE areas). Because the differences in the duration of the intervention were nested within regions, controlling for regions also accounted for differences in the duration of the intervention. Stata's post-model estimation commands were used in estimating the difference-in-differences effect. For further explanation of this approach (39-40).

**Table 2.** Sample-sizes, by region, time period, and group

Children aged 0-11 month(s)	SNNP (N)	SNNP (No. of clusters)	Amhara (N)	Amhara (No. of clusters)	Oromia (N)	Oromia (No. of clusters)	Total (N)	Total no. of clusters
Baseline (control)	479	41	298	30	305	30	1,082	101
Baseline (intervention)	418	23	300	30	318	30	1,036	83
Endline (control)	289	30	302	30	305	30	896	90
Endline (intervention)	310	30	300	30	305	30	915	90
Total	1,496	124	1,200	120	1,233	120	3,929	364
Children aged 12-23 months	SNNP (N)	SNNP (No. of clusters)	Amhara (N)	Amhara (No. of clusters)	Oromia (N)	Oromia (No. of clusters)	Total (N)	Total no. of clusters
Baseline (control)	465	41	300	30	309	30	1,074	101
Baseline (intervention)	424	23	300	30	292	30	1,016	83
Endline (control)	298	30	300	30	305	30	903	90
Endline (intervention)	301	30	299	30	311	30	911	90
Total	1,488	124	1,199	120	1,217	120	3,904	364
Women aged 15-49 years	SNNP (N)	SNNP (No. of clusters)	Amhara (N)	Amhara (No. of clusters)	Oromia (N)	Oromia (No. of clusters)	Total (N)	Total no. of clusters
Baseline (control)	480	41	300	30	297	30	1,077	101
Baseline (intervention)	420	23	300	30	296	30	1,016	83
Endline (control)	300	30	300	30	303	30	903	90
Endline (intervention)	300	30	300	30	304	30	904	90
Total	1,500	124	1,200	120	1,200	120	3,900	364

### Ethical approval

Each RHB issued an official letter of ethical approval for the surveys. The letter was stamped by officials at the *kebele* level. No obstacles to ethical approval of the research were encountered. At the household level, the investigators showed the stamped letter to the interviewee or head of household and read the consent form for participation in the survey. The respondents were informed that their answers would remain anonymous in the data-entry and reporting process.

## RESULTS

The baseline characteristics (Table 3) show demographic similarity between the comparison and intervention groups.

Women in the intervention group were slightly older than those in the comparison group, with some regional variation in maternal education. In SNNP, there were slightly higher proportions of women with no education in the comparison area compared to the ESHE area. In Oromia, women in the ESHE Project area were less educated compared to those in the control areas. The all-ESHE sample showed very similar demographics in the intervention and comparison areas even though the *woredas* were not randomly selected before assignment. Comparison of outcome indicators at baseline found no systematic pattern, suggesting that the possibility of selection bias was low.

Table 4 presents results from the SNNP, Amhara, and Oromia regions.

In SNNP, the regression results showed significant improvement in latrines and vitamin A supplementation in the ESHE area, after controlling for confounders (footnote of Table 3) and differencing out changes in the comparison area. In Amhara, only MCPR showed a significant change from baseline to endline. The most significant changes occurred in the Oromia region where latrines, DTP3 and measles coverage, and vitamin A supplementation were significantly higher at the endline in the ESHE project area. EBF showed large net percentage increases in SNNP and Oromia; however, the significance of the results fell just outside the 95% confidence interval, in part, due to decreased power to detect change in the 0-5 month subsample.

Table 5 presents difference-in-differences results for all ESHE intervention areas in the three regions compared to the controls.

The total differenced effects of ESHE on latrines, DTP3 and measles coverage, and vitamin A supplementation were significant (p<0.05). Total differenced effects of ESHE on EBF and MCPR were not significant (p>0.05).

## DISCUSSION

There were significant increases across multiple indicators. Improvements in four of the six indicators across the three regions were driven by a strong pattern of acceleration in Oromia region. Oromia has the largest population and geographic size of the three regions. One explanation for greater relative change between intervention and non-intervention areas in Oromia could be that ESHE spill-over was less pronounced due to lack of geographic proximity. Another contributor could have been the strong team of RHB and ESHE staff in Oromia, enhancing leadership and strengthening implementation.

In SNNP, despite impressive increases across both intervention and non-intervention areas in all six indicators, the intervention areas out-performed in only two. Because ESHE started first in SNNP and covered a larger proportion of *woredas*, there was likely a higher spill-over effect, which may have contributed to stronger overall regional increases and a lower differential between intervention and non-intervention areas. SNNP out-performed the other two regions in latrines regionally, with a significant booster effect in ESHE areas. The SNNP's RHB advocated vociferously for latrine construction and awarded prizes at *woreda* and *kebele* levels during annual reviews. ESHE may have influenced increased vitamin A supplementation in SNNP intervention areas by assisting the RHB to advocate for vitamin A supply and generating demand via community health promoters.

**Table 3.** Description of comparison and intervention groups

Variable	Characteristics	SNNP only, baseline (2003)		Amhara, baseline (2003)		Oromia, baseline (2003)		All ESHE, baseline (2003)	
		Comparison (%)	Intervention (%)	Comparison (%)	Intervention (%)	Comparison (%)	Intervention (%)	Comparison (%)	Intervention (%)
Maternal education	No education	67	55	79	80	70	88	72	71
	Primary	26	33	13	15	19	8	20	21
	Secondary+	7	12	8	5	11	4	8	8
Time to health centre	Less than 1 hour	44	50	40	55	41	23	42	45
	1-2 hour(s)	43	40	45	37	42	59	43	44
	2+ hours	13	10	15	8	17	18	15	11
Age of mother (completed years)	15-24	31	21	39	33	43	45	36	31
	25-34	51	62	44	40	44	41	47	50
	35-49	18	17	17	27	13	13	17	19
Age of child (months)	0-11	47	46	49	49	46	49	76	77
	12-23	53	54	51	51	54	51	24	23
Sex of child	Male	52	53	53	48	52	53	52	51
	Female	48	47	47	52	48	47	48	49
Access to clean water	Drinking-water from piped source of covered well/spring	67	67	61	50	52	35	62	54
Marital status	Married	94	97	89	84	94	95	93	92
Total N=4,747		1,076	948	683	738	646	656	2,405	2,342

Categories will not always add up to 100% due to rounding

**Table 4.** Difference-in-differences (DiD), regional results: SNNP, Amhara, and Oromia*

Region/ Group/ Time	SNNP						Amhara						Oromia					
	ESHE		Non-ESHE		DiD** Net % point change	N	ESHE		Non-ESHE		DiD** Net % point change	N	ESHE		Non-ESHE		DiD** Net % point change	N
	Baseline	Endline	Baseline	Endline			Baseline	Endline	Baseline	Endline			Baseline	Endline	Baseline	Endline		
Percentage of households that have latrines	35.1	89.5	32.8	76.5	10.7 (p=0.002)	1,477	28.8	45.6	27.3	43.7	0.4 (p=0.449)	1,198	19.8	63.9	38.6	50	32.7 (p=0.000)	1,194
Percentage of married women of childbearing age currently using modern family planning methods	17.8	36.2	12.6	27.7	3.3 (p=0.888)	1,404	14.1	31.8	17.4	22.5	12.6 (p=0.050)	1,028	15.6	32.1	20.7	31.7	5.5 (p=0.587)	1,107
Percentage of children aged 12-23 months, who received DPT3 vaccination	49.3	71.1	38.7	56.7	3.8 (p=0.335)	1,478	49	65.1	51.7	66.3	1.5 (p=0.757)	1,198	32.3	60.3	48.2	46.4	29.8 (p=0.000)	1,203
Percentage of children aged 12-23 months who received measles vaccination	49.3	69.2	45.2	57.7	7.4 (p=0.117)	1,485	45	63.1	52	64	6.1 (p=0.363)	1,199	43.3	59.4	60.7	42.9	33.9 (p=0.000)	1,205
Percentage of children aged 0-5 month(s) exclusively breastfed	54.3	72.4	50.2	55.9	12.4 (p=0.064)	769	79.5	81.1	78.2	87.8	-8 (p=0.179)	625	40.3	83	47.1	76.1	13.7 (p=0.173)	656
Percentage of children aged 6-23 months receiving vitamin A supplementation	17.1	64.6	29.9	60	17.4 (p=0.000)	2,049	13.8	70.1	17.6	75.1	-1.2 (p=0.925)	1,735	39	75	56	78.9	13.1 (p=0.022)	1,754

*Controls for EBF, DTP3, measles, and vitamin A were: sex of the child, household time to health centre, and maternal education. Modern CPR and latrines (controls) included: maternal age, education, water source, and household time to health centre;

**DiD is calculated as (Intervention Endline %–Intervention Baseline %)–(Com-parison Endline %–Comparison Baseline %). Results displayed represent two steps in the analysis of data: the significance, defined by a p value <0.05, represents the results from the multivariate logistic regression on the time-group interaction variable, which is the key independent variable of a DiD regression. Since the interaction coefficient is non-intuitive, we have, instead, depicted the difference over time between the intervention and non-intervention groups, using the predicted probabilities resulting from the regression. Essentially, this is the net percentage point change in the ESHE region once the comparison group change is subtracted

In Amhara, only MCPR showed a significant increase in the intervention areas compared to non-intervention areas. Immunization was a regional priority evident in higher performance at endline across intervention and non-intervention areas. There was no known wide-scale support for EBF in non-intervention areas to explain the paradoxical higher levels recorded in non-intervention areas at endline. The ESHE and RHB teams both faced personnel challenges in Amhara, which may have affected management and performance.

The Government's decentralization process deepened throughout ESHE. Although the FMOH established policy, each region developed their own priorities and strategies to achieving these. The ESHE Project supported this government-led process. The regions varied in how programmes were prioritized, contributing to the mixed results by region and programme. Despite efforts to phase-in interventions, regions struggled to sustain performance across multiple programmes when taken to scale.

Ethiopia is a large country with multiple health partners. Isolating impact of one partner, using baseline and endline surveys, is challenging. The combination of projects working at various scales challenges researchers’ ability to strictly segregate *woredas* into ‘intervention’ and ‘non-intervention’ areas. The ESHE Project focused on management, planning, training, supervision, and community mobilization support in interventions to improve the six indicators selected. Improved outcomes required important inputs from partners in addition to the Government's inputs, notably UNICEF for vaccines and vitamin A, and USAID and UNFPA for contraceptives. As noted in the methods, as with any quasi-experimental design, there are limitations in our findings. The major limitation of the study is the possible selection or programme placement bias. Accordingly, the effects of ESHE could be overestimated if selection of intervention areas were systematically associated with areas with relatively higher levels of other developmental factors that also influenced child health outcomes. Similarly, if selection of ESHE areas were systematically associated with areas with relatively lower levels of other developmental factors, that also influenced child health outcomes—this would potentially result in no effect or even a negative effect of ESHE. The comparison of child health outcomes during the baseline showing no difference, however, indicates that the presence of such selection bias was less likely.

**Table 5.** Difference-in-Differences (DiD) All ESHE Results*

Group/Time	ESHE		Non-ESHE		DiD** Net % point change	N
	Baseline	Endline	Baseline	Endline		
Percentage of households that have latrines	29	66.2	32.5	57.1	12.6 (p=0.002)	3,835
Percentage of married women of childbearing age cunently using modern family planning methods	16.2	33.6	16	27.4	6 (p=0.230)	3,538
Percentage of children aged 12-23 months, who received DPT3 vaccination	44.8	65.3	44.6	56.7	8.4 (p=0.007)	3,879
Percentage of children aged 12-23 months, who received measles vaccination	46.3	63.9	50.8	55.5	12.9 (p=0.000)	3,885
Percentage of children aged 0-5 month(s) exclusively breastfed	59.6	78.3	57.2	73.7	2.2 (p=0.254)	2,070
Percentage of children aged 6-23 months, who received vitamin A supplementation	22.6	69.9	33.3	70.8	9.8 (p=0.000)	5,534

*Controls for EBF, DTP3, measles, and vitamin A were: sex of the child, household time to health centre, and maternal education. Modern CPR and latrines (controls) included: maternal age, education, water source, and household time to health centre. The All ESHE analysis included regional effects;

**DiD is calculated as (Intervention Endline %-Intervention Baseline %)-(Comparison Endline %-Comparison Baseline %). Results displayed represent two steps in the analysis of data: the significance, defined by a p value <0.05, represents the results from the multivariate logistic regression on the time-group interaction variable, which is the key independent variable of a DiD regression. Since the interaction coefficient is non-intuitive, we have, instead, depicted the difference over time between the intervention and non-intervention groups, using the predicted probabilities resulting from the regression. Essentially, this is the net percentage point change in the ESHE region once the comparison group change is subtracted

In addition to aforementioned spill-over effects due to proximity of ESHE and non-ESHE clusters, the differenced effect of ESHE may also be under-estimated due to intentional spill-over effect in ESHE's design. The Government shared improvements and innovations in intervention areas with other *woredas* in real time. The RHB requested a design that would maximize the benefit of ESHE assistance for the entire region. The ESHE staff members were housed in the government zonal health offices, working with zonal health staff with the intention to motivate and build technical capacity, and zonal staff members were trained as trainers in all interventions. With other funding, zonal staff printed additional copies of training materials and took the training to non-intervention *woredas*.

While the surveys captured a good amount of information, some key controls were not collected, such as wealth or expenditure data, nor were complete information available on mothers for every sampled child (noted previously). Our approach to imputing the latter, using cluster means, introduces increased intra-cluster correlation. As a result, the standard errors are likely to be somewhat low and the significance of project effects could be overestimated (41). Sampling methodology could have been further strengthened by increasing the sample-size to increase the power, particularly of subpopulations, such as infants aged 0-5 month(s), to measure EBF. Post-hoc power analysis indicated that the sample for EBF in the ESHE intervention areas was low (0.22) but was adequate (above 0.8) for the remaining outcomes.

These issues noted that the ESHE was one of the few large-scale, field-based projects to rigorously document its effect. The Project's success in improving several child health practices, particularly in Oromia region, has been recognized by the FMOH, and the Project was scaled up to a fourth region—Tigray. This follow-on project increased the total number of *woredas* to 278, covering over 35 million people, and integrated evidence-based maternal, newborn and child health interventions, including nutrition, immunization, family planning, and integrated community case management through HEWs. The FMOH adopted a new policy in December 2010 to allow HEWs to manage pneumonia with oral antibiotics. National scale-up of iCCM (diarrhoea, malaria, pneumonia, and acute severe malnutrition) by HEWs began in 2011. The 2011 Ethiopia DHS (42) reports significant improvements from 2005 to 2011 in under-five mortality, stunting, undernutrition, and immunization coverage.

### Conclusions

Despite a probable spill-over effect that may have detracted from ESHE's full influence, we conclude that ESHE contributed to accelerated coverage of child health practices through its support to scale-up of evidence-based child health interventions. Any success of ESHE in contributing to these results likely comes from the integration of these initiatives to improve health workers' performance, strengthen health systems, and engage communities. The ESHE Project influenced approaches of the FMOH child health programming and was among the several partners that assisted Ethiopia to improve child health practices.

## ACKNOWLEDGEMENTS

The ESHE Project was funded by USAID and implemented by John Snow, Inc., in collaboration with Abt Associates, Inc., the Academy for Educational Development and Initiatives, Inc. The authors gratefully acknowledge the close collaboration provided by the FMOH of Ethiopia, RHBs, and communities of Amhara, Oromia, and SNNP regions. The household surveys were contracted to Mela Research, PLC, which was responsible for all aspects of the evaluation surveys, including report writing and presentation at national workshop. Their contract was financed using USAID project funding.

**Conflict of interest:** The authors have no conflicts of interest to disclose.
